# Virtual Reality Biofeedback in Health: A Scoping Review

**DOI:** 10.1007/s10484-021-09529-9

**Published:** 2021-12-03

**Authors:** Robin Lüddecke, Anna Felnhofer

**Affiliations:** 1 Department of Pediatrics and Adolescent Medicine, Division of Pediatric Pulmonology, Allergology and Endocrinology, Medical University of Vienna, Waehringer Guertel 18-20, 1090 Vienna, Austria; 2 Comprehensive Center for Pediatrics, CCP, Medical University of Vienna, Vienna, Austria

**Keywords:** Biofeedback, Virtual reality, Mental health, Stress, Psychophysiology, Motivation

## Abstract

Over the past decades, virtual reality (VR) has found its way into biofeedback (BF) therapy programs. Using VR promises to overcome challenges encountered in traditional BF such as low treatment motivation, low attentional focus and the difficulty of transferring learnt abilities to everyday life. Yet, a comprehensive research synthesis is still missing. Hence, this scoping review aims to provide an overview over empirical studies on VR based BF regarding key outcomes, included samples, used soft- and hardware, BF parameters, mode of application and potential limitations. We systematically searched Medline, PsycINFO, Scopus, CINAHL, Google Scholar and Open Grey for empirical research. Eighteen articles met the inclusion criteria. Samples mostly consisted of healthy (44.4%) and/or adult (77.7%) participants. Outcomes were mainly anxiety (44.4%), stress (44.4%) or pain reduction (11.1%), which were reduced by the VR-BF interventions at least as much as by classical BF. Participants in VR-BF interventions showed higher motivation and involvement as well as a better user experience. Heart rate or heart rate variability were the most frequently used BF parameters (50.0%), and most VR-BF interventions (72.2%) employed a natural environment (e.g., island). Currently, there is no clear evidence that VR-BF is more effective than traditional BF. Yet, results indicate that VR-BF may have advantages regarding motivation, user experience, involvement and attentional focus. Further research is needed to assess the specific impact of VR and gamification. Also, testing a broader range of clinical and younger samples would allow more far-reaching conclusions.

**Supplementary Information:**

The online version contains supplementary material available at 10.1007/s10484-021-09529-9.

## Introduction

Biofeedback (BF) may be defined as a process which enables individuals to intentionally alter their physiological activity (Schwartz, 2010). Precise instruments measure physiological signals (e.g., heart rate, breathing, muscle tone, or skin temperature) and simultaneously feed this information back to the individual via visual or auditory channels. Ultimately, the individual learns to become independent of the external feedback and progressively experiences control relying solely on internal feedback (Gaume et al., 2016). Areas of application for BF include the improvement of athletic, cognitive, or artistic performance (see Lehrer et al., 2020) and the treatment of a range of health conditions like chronic headache (Nestoriuc et al., 2008), pain (Sielski et al., 2017), high blood pressure (Nakao et al., 2003), or psychiatric disorders (Schoenberg & David, 2014) like anxiety disorders (Tolin et al., 2020) and depression (Walker & Lawson, 2013). Furthermore, BF may be used as a tool for managing stress and anxiety (Goessl et al., 2017).

Although extant data generally supports the use of BF for certain indications, there are several indications which may impact learning and outcome. For instance, it has repeatedly been criticized that visual representations of physiological signals are too abstract, too complex, or not meaningful enough to the user (Blum et al., 2019; Yu et al., 2018). Similarly, the task content has frequently been labelled as “extremely boring” (Gaume et al., 2016, p. 907), and has been found to lack the ability to sufficiently engage the user’s attention (Blum et al., 2019).

According to the psychoengineering model by Gaume et al. (2016) the following five key mechanisms shape BF learning: perceptibility, autonomy, mastery, learnability, and motivation. Particularly with regards to motivation, the authors see considerable need for improvement. It has been shown that extrinsic feedback (e.g., scores, money) facilitates the learning process. Hence, keeping the patient sufficiently motivated and installing adequate reward systems are among the main challenges to training success (see also Schwartz, 1973). In sum, participants must take an active role and continue practicing in order to develop a desired skill (Frank et al., 2010). Apart from motivation, sufficiently engaging and keeping up the users’ attention during the task may be regarded another key factor in the success of BF (Blum et al., 2019). Learning to self-regulate requires the user to keep a sustained attentional focus on the feedback signal. Yet, unappealing task content and distractions from the (laboratory) surroundings, as well as disrupting thoughts may all hinder the user to continuously focus on the task at hand. Another issue lies in the predictive validity of BF tasks (Gaume et al., 2016). The main goal of BF is that users increasingly acquire autonomy in the sense that they learn to rely on their internal feedback. These newly learnt skills then need to generalize from the abstract BF training to the more complex and challenging realm of everyday life. Yet again, abstract stimuli and a rather sterile training environment may make this generalization difficult.

### Virtual Reality and Gamification

Embedding BF in a virtual reality (VR) environment may constitute a viable solution to the above-mentioned challenges. Virtual reality is defined as an advanced human–computer interface that simulates a realistic virtual environment (VE) and allows participants to interact with it (Latta & Oberg, 1994). Using interactive elements and multisensory stimulation, VR manages to induce considerable levels of excitement and involvement (Maarsingh et al., 2019).

Additionally, VR facilitates the integration of gamification elements such as progression systems, story lines, or incentives (Deterding et al., 2011). Gamification approaches have experienced a rapid adoption in different scientific disciplines like education, health or work (Hamari et al., 2014). So called serious games combine gamification with educational or therapeutic purposes (Connolly et al., 2012) and have repeatedly been found to be effective in improving motivation (Domínguez et al., 2013; Sailer, 2016). Hence, using gamification mechanics in connection with BF promises to overcome the challenge of low motivational engagement. This may especially be true for children and adolescents. Past findings (see the meta-analysis of Fadhli et al., 2020) indicate that gamification can successfully be used to effectively engage younger generations in learning.

Furthermore, the use of immersive VR technology such as head-mounted-displays (HMDs), which cover the users’ full field of view (Slater & Wilbur, 1997), may promote sustained attention. Prior research has suggested that particularly immersive and interactive VEs which depict a nature environment like a beach, or a mountain scenery are effective in terms of stress reduction and relaxation (e.g., Annerstedt et al., 2013; Liszio et al., 2018). Virtual nature environments may be particularly suitable for BF applications as they can “replenish attentional resources in a comforting and relaxing way and provide plenty of opportunities for immersive and meaningful feedback elements” (Blum et al., 2019, p. 3). In their complexity and interactivity, VEs may furthermore improve the generalization of learnt skills to real-world settings. Generally, VEs are regarded as more ecologically valid than abstract tasks presented via 2D-screens (see Kothgassner & Felnhofer, 2020). Even when depicting phantasy worlds (e.g., underwater surroundings), VEs provide complex, contextually rich scenarios whose sensory vividness approximates that of everyday environments; also, the dynamic engagement of the sensorimotor system provokes more naturalistic behavioral and physiological responses than abstract stimuli (Bohil et al., 2011). According to this, the generalization to everyday life is expected to be easier with VR-BF than with traditional BF (Pallavicini et al., 2009).

In light of these advantages, it is not surprising that VR has found its way into the field of BF. However, VR based BF protocol are still rather new, and although the body of literature continues to grow, there is, to date, no research synthesis on VR-BF. Hence, we set out to conduct a scoping review to map out this emerging field with regards to the extent, range, and nature of existing literature to guide the planning and commissioning of future studies. In particular, we were interested in identifying key characteristics of existing studies such as included samples, design, types of physiological signals used, task content, type of VR equipment (hard- and software), mode of application (e.g., free body movements), and key outcomes (e.g., health, usability, motivation), as well as potential limitations (e.g., cybersickness).

## Methods

Since this research is rather young and heterogeneous, we chose the method of a scoping review to stake out the field (Arksey & O'Malley, 2005). Scoping reviews aim to provide an orientation over an ambiguous and heterogeneous field of research which is conceptually and methodologically too broad for a systematic review; another goal of scoping reviews is to create a knowledge synthesis of the main concepts and theories of a given field and map out open research questions (Tricco et al., 2018). Their objectives typically encompass summarizing research findings and identifying research gaps to derive recommendations for future studies (Peters et al., 2015). In our approach, we adhered to the methods proposed by Arksey and O'Malley (2005), and Levac et al. (2010); also, we closely followed the PRISMA-ScR Guidelines formulated by Tricco et al. (2018).

### Inclusion and Exclusion Criteria

We included studies in English and German from the beginning of database records until June 2021. Studies were eligible for inclusion if they evaluated a VR-BF treatment in at least one experimental group. We therefore limited our search to empirical research and excluded theoretical research like conceptual works. Furthermore, we excluded research that was solely qualitative, and reported no quantitative analyses. We searched peer reviewed and grey literature, and no limit was imposed on age groups. Our search exclusively focused on BF methods using peripheral psychophysiological parameters (i.e., heart rate (HR), heart rate variability (HRV), electrodermal activity (EDA), and breathing). Neurofeedback as well as proprioceptive and motor feedback interventions were excluded from the current scoping review for the sake of more stringency in content. In accordance with the VR definition of Riva (2003), we focused on studies utilizing immersive HMDs instead of flat screen or room-based systems.

### Search Strategy

We searched the databases Medline, PsycINFO (OVID), Scopus, CINAHL, Google Scholar and Open Grey for fitting literature. Additionally, Google Scholar alerts were enabled to ensure inclusion of articles in press. Search terms were selected to target papers addressing the intersection between BF (biofeedback, biofeedback training) and VR (virtual reality, vr, virtual environment, computer simulation). A detailed list of search strings is available as a supplement (Appendix Table A).

### Study Selection and Data Extraction

We de-duplicated the retrieved records using Zotero v5.0.87. We then individually reviewed the titles and abstracts and removed those which did not fit the inclusion criteria. One author (R.L.) independently reviewed all remaining full texts to establish eligibility. Whenever he was unsure, consensus with the second author (A.F.) was sought. The reference lists of chosen full texts were searched manually to find studies which had not been identified in the database research. To ‘chart’ the data, we built a form that we tested on articles we had retrieved in an earlier prescreening. Charting is a technique “for synthesizing and interpreting qualitative data by sifting, categorizing, and sorting material according to key issues and themes” (Arksey & O’Malley, 2005, p. 15). Following this technique, we charted key information and quantitative data in the full texts. We used Microsoft Excel to design a data charting form based on the variables relevant with regards to the scope of the review.

## Results

**Fig. 1:**
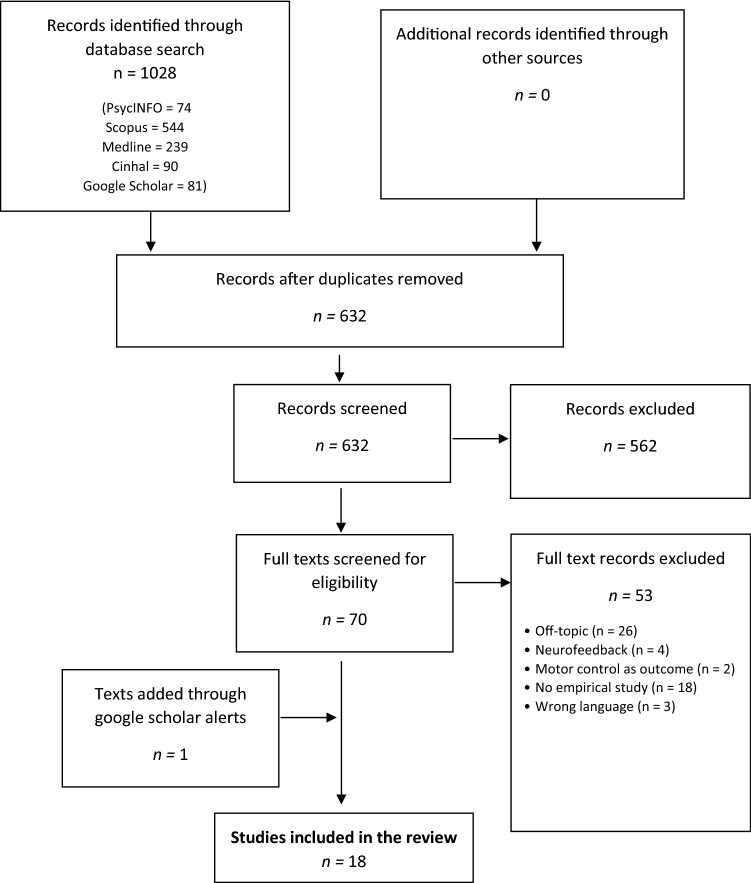
PRISMA flowchart of screening, exclusion, and inclusion criteria

Our initial search yielded 1028 articles. After removing duplicates and papers not fitting the inclusion criteria, n = 18 studies with 865 subjects (54.54% female; of those studies which reported gender) were included in this review (Table 1). For the detailed selection process, see Fig. 1. Most studies (n = 13) were published between 2016 and 2021. Studies originated from different countries, yet most studies could be related to either Germany (n = 5), the Netherlands (n = 5) or Italy (n = 4). Studies either reported randomized controlled trials (RCTs, n = 9) or a pre-post-design (n = 9). For the studies’ place of origin, objectives, design, used methods, measures and outcomes see Table 1.

**Table 1:** Characteristics of studies included in this review

Reference Country	Application	Sample	Participants (% female) age (range)	Design	Sessions (duration)	Intervention	Type of feedback	Outcomes (measures)	Main findings
Blum et al. (2019), Germany	*Sunset beach* freeing the sky from clouds, lighting up campfires and lamps	Healthy	n = 60 (n/a) age: M = 33.5 SD = 9.4	RCT	1 (10 min)	VR + BF *vs.* Desktop PC + BF	*HRV* Dynamic change of cloud coverage, campfire	Stress (STAI-S) Relaxation (STAI-S) Relaxation efficacy (self-constructed items) Mind wandering (CIQ) Focus on present moment (SMS) Attentional resources (modified Stroop task)	VR-BF and traditional BF comparable in terms of BF performance VR-BF buffered perceived stress in subsequent stressor task, increased relaxation self-efficacy more, reduced mind-wandering, helped participants focus on the present moment, and helped preserve attentional resources
Bossen-brooek et al. (2020), Netherlands	*DEEP* exploring an underwater world	ADHD/ ASD	n = 8 (12%) age: M = 14.67 SD = 1.83 (12.94–17.34)	Single-case study	6 (12.41 min)	VR + BF	*Breathing* Dynamic change of circle that corresponds to in-& exhalation, movement control	Anxiety (STAI) Disruptive classroom behavior (teacher interviews)	Small significant reduction in anxiety Small non-significant reduction in disruptive classroom behavior
Gaggioli et al. (2014), Italy	*Interreality Project* tropical island with relaxation areas (campfire, beach, waterfall)	Stressed	n = 121 (n/a) age: M = 42.96 SD = 9.32	RCT	10 (60)	VR + BF + Smartphone (IR) vs Classical CBT vs. waitlist	*HR/HRV* Dynamic change of fire intensity, movement of waves, movement of water	Trait Anxiety (STAI) Coping (COPE) Stress (PSS, PSM) Satisfaction with life (SWLS)	CBT and IR significantly reduced perceived stress, only IR significantly reduced trait anxiety CBT and IR significantly improved coping skills, IR significantly greater increase No difference in Satisfaction with life
Gorini et al. (2010)^b^, Italy	*INTREPID Project* tropical island with relaxation areas (campfire, beach, waterfall)	GAD	n = 20 (n/a) age: n/a (18–50)	RCT	8 (n/a)	VR + smartphone + BF *vs.* smartphone + BF *vs.* waitlist	*HR* Dynamic change of fire intensity, movement of waves, movement of water	GAD (EDA, HR, STAI-Y, HAM-A)	GAD improvement in both experimental groups post treatment Only in VR-smartphone BF group significant reduction in STAI-Y-scores post treatment
Hendriks and Rombout (2018), Netherlands	*Calm Forest* changing environmental light	Healthy	n = 12 (41%) age: n/a	Single-case study	1 (n/a)	VR (HQ) + BF *vs* VR (HQ) + no BF *vs.* VR (LQ) + BF *vs* VR (LQ) + no BF	*DB* Dynamic change of environmental light	State Anxiety (SUDS)	State Anxiety tended to decrease non-significantly over all conditions In the no-BF-Condition State Anxiety non-significantly higher in high-quality condition than in the low-quality condition
Houzangbe et al. (2020), France	*Futuristic laboratory rooms* succession of rooms with different BF tasks each	Healthy	n = 30 (26%) age: M = 25.87 SD = 5.237 (21–43)	Single-case study	1 (12 min)	VR + BF	*HR* Dynamic change of lights or lasers	Agency Perceived usability Felt involvement Focused attention Personal gratification (self-constructed items)	Significantly higher involvement and personal gratification, No significant difference in perceived usability, focused attention and agency compared to the average score
Kojić et al. (2019), Germany	*Rowing simulator* static mountain lake picture with an overlay of a physiological signal	Healthy	n = 23 (43.5%) age: M = 23.67 SD = 4.887 (17–36)	Within-subjects-design	5 (60 min)	VR + BF as line chart *vs* VR + BF as lung animation *vs* VR + all visualiza-tions of BF *vs* no VR, no BF *vs* VR + no BF	*RR* Physiological signal (breathing as line chart or lung animation)	Breathing pattern (RR) Flow (SFSS-2, IPQ) Sympathy Helpfulness	Correct RR decreased for conditions with visualizations in VR User experience enhanced through VR Flow, sympathy, and helpfulness higher in all VR conditions
Maarsingh et al. (2019), Netherlands	*Stressjam* tropical island	(1) Stressed (2) Healthy	(1) n = 64 (51.6%) age: M = 40.6 SD = 11.5 (2) n = 111 (62.2%) age: M = 43.0 SD = 10.5	Case control study	(1) 3 (60 min) (2) 1 (60 min)	(1) VR + BF (2) VR + BF	*HRV* Dynamic change of visual cues based on HR (i.e. door opens with low HR)	Stress (HRV) Stress mindset (SMM-G) Personal involvement (PII) System usability (SUS)	﻿The healthy participants and the patient sample both had a more positive stress mindset after using the application than at baseline
Pallavi- cini et al. (2009)^b^, Italy	*INTREPID Project* tropical island with relaxation areas (campfire, beach, waterfall)	GAD	n = 12 (75%) age: M = 47 SD = 11.92^a^	RCT	8 (n/a)	VR + Smartphone + BF (VRMB) *vs* VR + Smartphone (VRM) *vs* Waiting list	*HR* Physiological signal (bar with HR) Dynamic change of visual cue ((a)fire intensity, (b) move-ment of waves, (c) movement of water)	GAD (EDA, HR, STAI-Y, BAI, PSWQ, VAS-A)	Only in VRMB group significant anxiety reduction after treatment ﻿Tendency indicating a decrease in HR and EDA between pre- and post-session in the VRMB group, higher than in the VRM
Prabhu et al. (2019), USA	*Virtual beach* clearing the beach of fog	Total knee athroplasty	n = 5 (60%) age: M = 68 SD = 3.4	Single-case study	1 (n/a)	VR + BF	*HRV, RR* Physiological signal (HRV-curve) Dynamic change of visual cue (fog on beach)	Anxiety (n/a) Pain (n/a)	27.6% pain reduction 33% anxiety reduction after intervention compared to base results
Repetto et al. (2011)^b^, Italy	*INTREPID Project* tropical island with relaxation areas (campfire, beach, waterfall)	GAD	n = 24 (n/a) age: M = 47.8 SD = 12.18 (18–50)^a^	RCT	8 (n/a)	VR + Smartphone + BF (VRMB) *vs* VR + Smartphone (VRM) *vs* Waiting list	*HR, EDA* Physiological signal (bar with HR) Dynamic change of visual cue ((a)fire intensity, (b) movement of waves, (c) movement of water)	GAD (STAI-Y, HAM-A)	Significant reduction in physiological and self-assessed measures of anxiety at the end of the different sessions; tendency of higher HR and EDA decrease in experimental group
Rockstroh et al. (2021), Germany	*Flowborne* relaxing ruins and forest environments	n/a	n = 45 (64.4%) age: M = 22.9 SD = 5.4	Single-case study	6 (8 min)	VR + BF	*DB* Dynamic change of visual cue (changing colors in the environment)	User experience (self-constructed items) Momentary relaxation (single item) Perceived stress (PSS-10) Burnout (CBI) Self-efficacy (self-constructed items)	Increased use of DB, significant reduction of perceived stress and burnout, no differences in work-related burnout symptoms, significantly increased relaxation-related self-efficacy
Rockstroh et al. (2020), Germany	*Forest* dynamic weather conditions	Healthy	n = 94 (68%) age: M = 23.8 SD = 4.9	RCT	1 (10 min)	VR + BF *vs* VR w/o BF *vs* screen + BF *vs*.screen w/o BF	*EDA* Dynamic change of visual cue (weather condition) Dynamic change of Soundscape (weather)	Sense of presence (IPQ); Perceived stress (STAI-S) Restorativeness (PRS)	No treatment-specific differences in subjective stress or physiological arousal; increased sense of presence and in parts perceived restorativeness in EG
Rockstroh et al. (2019), Germany	*Sunset beach* freeing the sky from clouds, lighting up campfires and lamps	Healthy	n = 68 (51.6%) age: M = 22.9 SD = 4.0	RCT	1 (10 min)	VR-BF *vs* BF	*HRV* Dynamic change of visual clues (cloud coverage, campfire)	Mood (MDBF) Motivational aspects (self-constructed item(s)) Attentional focus (self-constructed item(s))	VR-HRV-BF and HRV-BF are equally effective in increasing short-term HRV VR-HRV-BF was associated with higher motivation and helped users better to maintain attentional focus
Tinga et al. (2019), Netherlands	*Mindful breathing* white cloud moving	Healthy	n = 60 (61.7%) age: M = 22.07 SD = 3.03 (18–31)	RCT	1 (6 min)	VR + BF *vs* VR + BF placebo *vs* VR w/o BF	*ECG, RR* Dynamic change of visual cue (cloud in front of face)	Subjective arousal (self-constructed item(s)) Objective arousal (ECG, EEG, RR) Subjective experience (self-constructed item(s))	Subjective and objective arousal decreased in all conditions. Respiratory BF had no additional value in reducing arousal
Tong et al. (2015)^b^, Canada	*Virtual Meditative Walk (VMW)* guided forest path	Chronic pain	n = 13 (53.8%) age: M = 49 SD = 8.2 (35–55)	RCT	1 (12 min)	Deepstream stereoscopic viewer + BF (audio track) *vs.* BF (audio track)	*EDA* Dynamic change of amount of fog in the woods	Pain reduction (self-constructed items)	VR + BF audio track significant more effective than BF (audio track) only in reducing reported pain levels
Tu et al. (2020), USA	*Breath Coach* moving a ball on a track, moving a circle	Healthy	n = 10 (n/a) age: M = n/a N = n/a	Single-case study	6 (15 min)	VR + BF *vs*.BF	*BP, IBI, RSA* Movement control through breathing	Stress reduction (HRV) Training experience (6-item self-report measure, BP)	BreathCoach seems more effective than traditional training when it comes to RSA maximization, cognitive function, and stress reduction Lower frequency of feeling distracted and anxious
Van Rooij et al. (2016), Netherlands	*DEEP* exploration of an underwater world	n/a	n = 86 (39.5%) age: M = 10.1 SD = 1.4 (8–12)	Single-case study	1 (7 min)	VR + BF	*DB* Dynamic change of visual cue (circle that corresponds to in- and exhalation), movement control through breathing	Self-reported state-anxiety (STAI-C) Self-reported positive and negative affect (STAI-C) Experience (self-constructed item(s))	Significant decrease in self-reported state-anxiety compared to baseline No significant differences in positive and negative affect compared to baseline

*ASI* Anxiety Sensitivity Index, *BAI* Beck Anxiety Inventory, *BP* Breathing pattern, *CBI* Copenhagen Burnout Inventory, *CBT* Cognitive behavioral technique, *CTH* Chronic tension headache, *CIQ* Cognitive Interference Questionnaire, *COPE* Coping Orientation to the Problems Experienced Inventory, *DB* Diaphragmatic breathing, *ECG* Electrocardiogramm, *EDA* Electrodermal activity, *GAD* Generalized anxiety disorder, *HAM-A* Hamilton Anxiety Rating Scale, *HMD* Head mounted display, *HR* Heart rate, *HRV* Heart rate variability, *IBI* Inter-beat interval, *IPQ* Igroup Presence Questionnaire, *MBSR* Mindfulness-based stress reduction, *MDBF* Multidimensional Mood Questionnaire, *PedsQL* Pediatric Quality of Life Inventory, *PII* Personal Involvement Inventory, *PRS* Perceived Restorativeness Score, *PSM* Psychological Stress Measure, *PSS* Perceived Stress Scale, *PSWQ* Penn State Worry Questionnaire, *RR* r rate, *RSA* Respiratory sinus arrhythmia, *SFSS-2* SHORT Flow State Scale, *SMM-G* Stress Mindset Measure, *SMS* State Mindfulness Scale, *STAI-C* State-Trait Anxiety Inventory for Children, *STAI-S* State-Trait Anxiety Inventory, *STAI-Y* State-Trait Anxiety inventory Form Y-2, *SUDS* Subjective Units of Discomfort Scale, *SUS* System Usability Scale, *SWLS* Satisfaction with Life Scale, *TAS-20* Toronto Alexithymia Scale, *TUI-BEN* Technology Usage Inventory, *VAS-A* Visual Analog Scala for Anxiety

^a^Calculated mean by authors of this study since the study itself only provide means divided by experimental groups

^b^Studies used the term GSR (galvanic skin response) instead of the currently used term EDA (electrodermal activity)

### Participants’ Characteristics

Overall, n = 14 studies used adult samples (> 18 years), only n = 2 studies were conducted among underage participants, and two (Hendriks & Rombout, 2018; Tu et al., 2020) did not report age. The mean age across all studies was M = 31.23 years, with the youngest sample being M = 10.1 years (Van Rooij et al., 2016) and the oldest sample being M = 68.0 years old (Prabhu et al., 2019). Most participants were healthy (n = 9 studies), followed by persons with general anxiety disorder (GAD) in n = 3 studies.

### VR technology and Implementation of Feedback

Most studies (n = 14) used virtual nature environments such as islands (n = 8), forests (n = 3), underwater worlds (n = 2), or a hilly landscape (n = 1). Also, the majority (n = 12) employed modern 100-to-110-degree diagonal field-of-view (FoV) HMD solutions, including the Oculus Rift or HTC Vive, while n = 4 studies used older HMDs like the VUZIX iWear, which are only capable of delivering a 30-to-40-degree FoV. One study reported the use of the smartphone-based HMD setup Google Daydream View (90 degree) and one used the Deepstream stereoscopic viewer (85 degree), a VR solution mounted on a mechanic arm instead of the participant’s head in order to free it from additional weight. Most studies (n = 15) were either single task exercises or a sequence of narratively unconnected tasks (e.g., calming down to lighten up a fire). Only n = 3 studies used a narrative logically connecting different exercises with each other, and only one embedded them into a larger storyline. All applications presented their feedback primarily visually; participants had to alter their physiological state in order to affect campfires, lamps, waterfalls or waves (n = 5), to change the weather (n = 6), or the color palette of the environment (n = 4), or to eliminate obstacles in the way (n = 2). Some used correctly performed BF as vehicle of movement through the VR (n = 3), and some presented just an animation like a flattening curve, a scale changing circle or a lung (n = 3). Only n = 2 studies also reported using audio output, and only one of them additionally changed the soundscape based on the physiological state. Eight studies used HR (n = 4) or HRV (n = 3) as the main BF parameter, followed by breathing (n = 5), and electrodermal activity (n = 2).

### Primary and Secondary Outcomes

A considerable number of studies (n = 8) targeted *anxiety* or *GAD* as primary outcomes. The authors consistently reported a significant anxiety-reducing effect of VR-BF compared to baseline. *Stress reduction* was evaluated in n = 6 studies; of those, five reported a significant effect compared to base level, and one found no treatment-specific differences in subjective stress or physiological arousal compared to controls (BF on a computer screen, and VR without BF). Furthermore, n = 3 reported a decrease in *pain* when compared to baseline or to treatment as usual (TAU) without VR or BF. Other findings regarding primary outcomes were a significantly improved *quality of life* (n = 1) and *coping skills* (n = 1), a non-significant reduction in *disruptive classroom behavior* (n = 1), and no changes in *self-reported and physiological arousal* compared to placebo (n = 1), as well as in positive/negative affect compared to baseline (n = 1).

Regarding secondary outcomes, n = 13 studies focused on user experience or involvement. Findings showed a higher sense of presence (n = 1), a significantly better training experience such as relaxed and stable respiration and sustained attention (n = 2), and higher motivation to use VR-BF (n = 1) in the experimental groups than in classical BF settings carried out on a desktop PC. Furthermore, an increased attentional focus on the present moment and less incidents of mind wandering (n = 1), and higher levels of flow (n = 1) were found when compared to traditional BF. Studies also reported high levels of personal gratification (n = 1), and high involvement in the application (n = 1). VR-BF was also rated as more sympathetic and helpful than a classical BF condition (n = 1). Detrimental outcomes were rarely reported. Only one study assessed simulator sickness and another one signs of nausea while performing the VR intervention: Rockstroh et al. (2021) reported low values for simulation sickness (M = 1.40, SD = 0.40, Likert scale from 1 to 5), Van Rooij et al. (2016) reported that 86% of participants did not show any signs of nausea, yet the authors do not discuss the remaining 14% that did. Prabhu et al. (2019) reported excluding a priori participants with simulation sickness, and four studies reported excluding patients with neurological diseases, migraine, headache, or vestibular abnormalities (Gaggioli et al., 2014; Gorini et al., 2010; Pallavicini et al., 2009; Repetto et al., 2011). Other detrimental outcomes did not find mention in any of the studies.

## Discussion

This scoping review aimed to establish the state of research regarding a novel form of BF, i.e., the integration of VR in traditional BF protocols. The main objective was to provide an overview over study design, samples, used soft- and hardware, and outcomes.

Overall, 18 studies were included in the current scoping review, most of which were published in the last five years. This rise in interest may be explained by the acceleration in the development of VR technology which followed the launch of the affordable Oculus Rift HMD in 2013. The rapid growth of the VR market since then (Chang & Chen, 2017) has resulted in a broader variety not only of providers but also of cheaper VR soft- and hardware (Hodgson et al., 2015). Big game engine companies like Epic Games or Unity Technologies have expanded their gaming engines with tools to design environments directly for VR, thus making it easier to program inexpensive, high-quality VR applications. Accordingly, most studies included in this review applied of high-end, usable, and comfortable to wear HMDs like Oculus Rift or HTC Vive that provide a 100-to-110-degree diagonal FoV immersion instead of more conservative alternatives like the VUZIX Eyewear (30-to-40-degree) or the DeepStream stereoscopic viewer (85 degree).

Most studies originated from one of three countries: Italy, Germany, or The Netherlands. While research from Italy, namely the EU-funded INTREPID (Gorini et al., 2010; Pallavicini et al., 2009; Repetto et al., 2011) and INTERSTRESS (Gagglioni et al., 2014) projects performed ground work in the field, new research mostly emerges out of one research group located in Freiburg, Germany (Blum et al., 2019; Rockstroh et al., 2019, 2020, 2021) and several research groups in The Netherlands (Bossenbroek et al., 2020; Hendriks & Rombout, 2018; Maarsingh et al., 2019; Van Rooij et al., 2016).

The studies included in this review primarily targeted anxiety, stress, and pain as their main outcomes. Results indicate that VR-BF successfully reduces anxiety, stress and pain compared to baseline. When compared to classical BF, VR-BF was equally effective in reducing stress (Blum et al., 2019; Rockstroh et al., 2019, 2020) and anxiety (Gagglioni et al., 2014; Gorini et al., 2010; Repetto et al., 2011). With regards to pain reduction, VR-BF seems to be even more effective than traditional BF (Tong et al., 2015), yet, with just thirteen participants in total, the generalizability of this study’s finding is limited. Moreover, one study (Tinga et al., 2019) found a higher reduction in arousal in a control group using only VR (a moving cloud automatically simulating breath) and no BF compared to a VR-BF group (moving cloud controlled by participant’s respiration), raising the additional question of the necessity of BF in relaxation interventions.

While at this point there is no unambiguous evidence that VR-BF is more effective than classical BF, the current review indicates that VR based BF protocols have advantages over traditional ones when it comes to motivation, user experience and involvement. Results show a better training experience, a high motivation to use VR-BF, as well as strong involvement, better focused and sustained attention, and high levels of flow. Similarly, in the reviewed studies, personal gratification was elevated, and the technology was found to be sympathetic and helpful. In light of these encouraging findings, one may cautiously conclude that combining VR with BF may support particularly those factors which – according to the psychoengineering model (Gaume et al., 2016) – are key to feedback learning and thus, to the success of BF.

However, the role of gamification in increasing motivation remains unclear. Most studies in this review used single task exercises and only two studies applied an elaborate gamification approach: Houzangbe et al. (2020) placed the participants in a sequence of adjoining futuristic laboratory rooms, with the subsequent rooms opening up after room-specific BF exercises had been solved (e.g., regulating their HR to shoot a weapon). Similarly, in “Stressjam”, the exercises were embedded in a cohesive story line in which the participants had to prevent an island from being destroyed by a volcano (Maarsingh et al., 2019). Both studies, however, lacked a control group and tested their prototype in no more than three sessions, precluding generalizations and conclusions with regards to long-term treatment effects.

Overall, not only gamification elements, but also the VE itself may influence motivation. The interactive nature of fully immersive VEs has been found to be particularly engaging and add to a rewarding user experience (Tcha-Tokey et al., 2018). Above all, however, the advantage of using VR in combination with BF seems to lie in the positive impact it has on attention: The increase in focused and sustained attention, and the decrease in mind-wandering incidents, as well as experiences of flow (Csikszentmihalyi & Csikzentmihaly, 1990) may all be attributable to the immersiveness of the technology which shields from distractions (Slater & Wilbur, 1997). Furthermore, according to the *Attention Restoration Theory* (Kaplan, 1995), nature environments – such as those which were used in most studies – facilitate replenishing attentional resources. Despite these positive results, some authors (Yu et al., 2018) argue that interactive immersive VEs coupled with BF displays may require even more (attentional) effort to engage in the feedback learning process. Hence, the exact relationship between VR-BF and cognitive remains to be established.

Overall, the positive effects of VR do not seem to translate into an advantage of VR-BF over classical BF in terms of effectiveness. One answer may be that the motivational aspects only come into play after several sessions. At the beginning, classical BF as well as VR-BF may both profit of the novelty effect, but over time the abstract, “boring” (Gaume et al., 2016) traditional BF tasks may make it difficult keep up attention and motivation. This assumption is supported by the fact that those studies which compared VR-BF with a classical intervention over several sessions also found a higher decrease in HR and EDA (Pallavicini et al., 2009; Repetto et al., 2011) and stress (Tu et al., 2020) and a higher increase in coping skills (Gagglioni et al., 2014) in the VR-BF condition. Similarly, trials with more than three sessions were accompanied by a more positive training experience (Tu et al., 2020), high involvement (Maarsingh et al., 2019) and a higher feeling of flow and sympathy (Kojic et al., 2019). However, nine studies in this review performed BF only once. Hence, the impact of repeated VR based BF training on long-term motivation and ultimately on treatment outcomes remains to be evaluated.

The majority of participants in this review were adults. This is noteworthy, since children and adolescents may particularly benefit from innovative BF protocols which use gamification and VR to increase involvement and motivation (see Fadhli et al., 2020). While adults may succeed with a goal orientation (e.g., pain relief), children are even more dependent on an intervention which facilitates intrinsic motivation (Kanfer, 1990). Generally, the use of VR in underage samples particularly for therapeutic interventions is still in its infancy (Kothgassner & Felnhofer, 2021). Thus, future research is encouraged to increasingly consider children and adolescents as possible target groups for VR-BF.

Apart from minors, clinical samples are also worth investigating. It was surprising to find that most studies (n = 9) in this review focused exclusively on healthy participants. Only GAD (Gorini et al., 2010; Pallavicini et al., 2009; Repetto et al., 2011) and chronic pain (Prabhu et al., 2019; Tong et al., 2015) patients were treated in more than one study. Rather innovative indications such as with ADHD or ASD patients (Bossenbroek et al., 2020) show promising improvements with VR-BF. However, VR-BF interventions in these samples primarily focused on stress regulation and relaxation rather than on alleviating symptoms specific to ADHD and ASD such as attention deficits, impulsivity and hyperactivity, or social deficits and restrictive behaviors. This is in line with the assumption, that BF training may be more useful as an adjuvant therapy rather than a first line treatment for some disorders.

## Limitations

The present review has several limitations which are common for scoping reviews. First, we did not perform a quantitative data synthesis, as our research question was broad in scope and the quality of most included papers was rather low, with only nine papers reporting RCTs. Hence, future systematic reviews and meta-analyses should pursue a much narrower research question than the one posed for this review. Also, we could not derive any conclusions regarding possible contraindications to VR-BF. Even though VR constitutes a promising technology, it may, in some cases, induce cybersickness (i.e., nausea, headache, tiredness or discomfort, (Mehrfard et al., 2019). Hence, patients with epilepsy, monocular vision, or vestibular impairments are not suitable for inclusion in VR based treatments. All studies stated that they had excluded participants with the above-mentioned impairments, but no study raised additional impairments or reported on incidents of cybersickness. Similarly, none of the studies discussed the issue of body position and movements during training: Since VR is mostly used in a standing position, this could cause artifacts in physiological signals. Overall, a more thorough documentation in future research is necessary.

## Conclusion

Recent years have brought forward research on VR-BF interventions aimed at treating anxiety, stress, and chronic pain. HMDs have superseded alternate, less functional VE solutions such as smartphones or heavy, unwieldy apparatuses like the stereoscopic viewers. Despite these developments, the field may still be regarded as new. Further RCTs with representative samples and higher training frequency are necessary to establish whether VR-BF is superior to classical BF.

Also, further research is needed to assess the specific impact of VR and gamification on motivation, attention, and user experience. This would help establish if the use of BF protocols based on gaming mechanisms constitute an advantage over more traditional protocols, particularly in those target groups that are considered hard to motivate such as children and adolescents (see Fadhli et al., 2020). Finally, ecologically valid VEs promise to facilitate the transfer learnt skills to real-world settings (Kothgassner & Felnhofer, 2020). None of the studies included in this review assessed whether the newly acquired abilities were transferred to everyday life. Focusing on this aspect would help future research determine whether the use of VR has added benefit compared to traditional BF protocols or whether it is better suited as a complementary therapy.

## Supplementary Information

Below is the link to the electronic supplementary material.

Supplementary file1 (DOCX 14 kb)

## Data Availability

The authors confirm that the data supporting the findings of this study are available within the article and its supplementary materials.
