# Supplementary material for: Virtual Reality Biofeedback in Health: A Scoping Review
Source: Appl Psychophysiol Biofeedback. 2021 Dec 3;47(1):1–15. doi: 10.1007/s10484-021-09529-9 (PMC8831282; doi:10.1007/s10484-021-09529-9)
Supplement: Supplementary file 1 — Supplementary file1 (DOCX 14 kb) [file 10484_2021_9529_MOESM1_ESM.docx]

**Article Title:** Virtual Reality Biofeedback in Health: A Scoping Review

**Journal Name:** Virtual Reality

**Author Names:** Robin Lüddecke & Anna Felnhofer^1^

**^1^Affiliation and E-mail of corresponding author:**

Department of Pediatrics and Adolescent Medicine, Medical University of Vienna,

Waehringer Guertel 18-20, 1090 Vienna, Austria,

e-mail: anna.felnhofer@meduniwien.ac.at

| **Supplement Table A1.** Databases and Search Terms Used | |
| --- | --- |
| Database | MESH/search terms |
| Medline | (biofeedback.mp. OR training, biofeedback.mp.) AND (virtual reality.mp OR computer simulation.mp. OR vr.mp OR virtual environment.mp) |
| PsycINFO | (biofeedback.mp. OR training, biofeedback.mp.) AND (virtual reality.mp OR computer simulation.mp. OR vr.mp OR virtual environment.mp) |
| Google Scholar | (biofeedback OR "training, biofeedback" OR "biofeedback training") AND ("virtual reality" OR "computer simulation" OR vr OR "virtual environment") -EMG  -neurofeedback -motor -proprioceptive |
| Scopus | (TITLE-ABS-KEY (biofeedback  OR  "training,biofeedback")  AND  TITLE-ABS-KEY ("virtual reality"  OR  "computer simulation"  OR  "vr"  OR  "virtual environment") ) |
| Cinhal | (biofeedback OR "training, biofeedback") AND ("virtual reality" OR "virtual environment" OR "vr" OR "computer simulation") |
| Open Grey | "biofeedback" OR "training, biofeedback" AND "virtual reality" OR "vr" OR "virtual environment" OR "computer simulation" NOT neurofeedback OR emg |
